# Pan-cancer analyses reveal cancer-type-specific autophagy signatures with potential implications for prognosis and therapy response

**DOI:** 10.7150/jca.101046

**Published:** 2025-01-01

**Authors:** Hongyan Yuan, Xiaojing Zhu, Jiaxing Zhang, Zixin Zhang, Weihua Li, Jiankai Xu

**Affiliations:** 1 College of Bioinformatics Science and Technology, Harbin Medical University, Harbin 150081, China.; 2 Medical imaging department, Shenzhen Second People's Hospital and the First Affiliated Hospital of Shenzhen University Health Science Center, Shenzhen 518035, China.

**Keywords:** Autophagy, Cancer, Prognosis, Tumor microenvironment, Therapy response

## Abstract

Autophagy is a common cellular degradation and recycling process that plays crucial roles in the development, progression, immune regulation, and prognosis of various cancers. However, a systematic assessment of the autophagy-related genes (ATGs) across cancer types is deficient. Here, a transcriptome-based pan-cancer analysis of autophagy with potential implications in prognosis and therapy response was performed. About 3 - 32 % of ATGs expressed differentially across 21 human cancers, and the autophagy-related score (ATS) based on differential ATGs could be used to predict the prognosis in 11 cancers, which was validated in multiple independent datasets. Autophagy was found to influence tumor immune microenvironment mainly by regulating tumor-infiltrating lymphocytes and myeloid-derived cells, and interactions between T cells and macrophages with lower ATS was enhanced to improve clinical outcomes by single cell analysis in bladder urothelial carcinoma (BLCA). In addition, the ATS was correlated with drug sensitivity and showed a capacity for prediction of therapy response in diverse cancers. Altogether, the results highlighted robust value of autophagy in cancer prognosis and treatment.

## Introduction

Autophagy, defined as a highly conserved catabolic process, maintains cell metabolism, genomic integrity, and cell survival through the degradation of cytoplasmic organelles, proteins, and macromolecules, and the recycling of the breakdown products 1-3. The dysfunction of autophagy has been associated with pathophysiological processes of cancer 4-6, which can either inhibit or promote tumor growth, depending on tumor type and stage, and the surrounding microenvironment 7, 8. Some studies have reported autophagy-related gene signatures in hepatocellular carcinoma 9, and bladder cancer 10, cervical cancer 11, but few of them have linked these autophagy signatures to mechanisms underlying tumor microenvironment. Therefore, there is a need to identify autophagy-related gene signatures with comprehensive analyses on their downstream effects in pan-cancer.

It has shown that autophagy-related genes (ATGs) are involved in the modulation of immune response 12. Noman *et al.* reported that hypoxia impaired elimination of non-small cell lung carcinoma cells by autologous cytotoxic lymphocytes 13. Deficiencies in certain autophagy regulators, such as ATG7, can lead to T cell dysfunction 14. The advent of single-cell RNA sequencing (scRNA-seq) technology provides a powerful tool to comprehensively explore the influence on the tumor microenvironment by autophagy. Tong *et al.* found that the effector function of T cells could be influenced and shaped by autophagy-associated pathways through the investigation of single-cell RNA sequencing in gastric cancer 15. In addition, Autophagy also plays an important role in cancer resistance 16. Autophagy modulation affects tumor resistance through some mechanisms that involve the PD1/PD-L1 axis 17 and CTLA4 signaling 18. Ahamed Saleem *et al.* reported that one type of autophagy inhibitors termed hydroxychloroquine was clinically beneficial for prostate cancer patients 19. Hence, connecting autophagy-related signatures to the TME and drugs will better understand the role of autophagy in the TME to overcome cancer therapy resistance.

In this study, we first performed an integrative analysis to explore the expression of 234 ATGs curated from human autophagy database (HADb) across 21 types of cancer. The RNA sequencing (RNA-seq) data from the gene expression omnibus database (GEO) database and the cancer genome atlas (TCGA) database were used for analysis and validation. We then used differential ATGs to establish autophagy-related score (ATS), and evaluated whether this signature was associated with survival outcomes and clinicopathological factors in 11 cancers. The association between ATS and tumor microenvironment was further investigated using bulk and single-cell RNA sequencing datasets. And finally, we evaluated the effects of ATS in identifying tumor immunotherapy and chemotherapy responders. Findings of this study may be valuable for predicting patients' prognosis and improving clinical therapeutic benefits.

## Materials and Methods

### Data collection and processing

HADb (http://www.autophagy.lu) is the first human autophagy-dedicated Database. Totals of 234 ATGs were downloaded from HADb as candidate genes. The expression data (RNA-sequencing read counts) and matched clinical data were downloaded from TCGA (https://portal.gdc.cancer.gov). We retained cancer types in which the number of cancer samples and normal samples is greater than 3. A total of 21 cancer types (bladder urothelial carcinoma (BLCA), breast invasive carcinoma (BRCA), cervical squamous cell carcinoma and endocervical adenocarcinoma (CESC), cholangiocarcinoma (CHOL), colon adenocarcinoma (COAD), Esophageal Carcinoma (ESCA), glioblastoma multiforme (GBM), head and neck squamous cell carcinoma (HNSC), kidney chromophobe (KICH),kidney renal cell carcinoma (KIRC), kidney renal papillary cell carcinoma (KIRP), liver hepatocellular carcinoma (LIHC), lung adenocarcinoma (LUAD), lung squamous cell carcinoma (LUSC), pancreatic adenocarcinoma (PAAD), pheochromocytoma and paraganglioma (PCPG), prostate adenocarcinoma (PRAD), rectum adenocarcinoma (READ), stomach adenocarcinoma (STAD), thyroid carcinoma (THCA), and uterine corpus endometrial carcinoma (UCEC)) were included in the analysis.

GSE2748, GSE13507, GSE68465, GSE72970, Breast Cancer (METABRIC, Nature 2012 & Nat Commun 2016), and ICGC-LIRI-JP were retrieved from Gene Expression Omnibus (GEO, https://www.ncbi.nlm.nih.gov/geo), cBio Cancer Genomics Portal (cBioPortal, http://www.cbioportal.org) and International Cancer Genome Consortium (ICGC, https://dcc.icgc.org), respectively. These datasets as independent external validation data, which comprised the gene expression and clinical data.

The scRNA-seq cohort of BLCA (GSE190888 and GSE186520) and KIRC (GSE121638) were downloaded from GEO. GSE103668 and GSE67501 contained cisplatin and bevacizumab treatment data for BRCA and nivolumab therapy information for KIRC, respectively. Cell line expression data and drug sensitivity data were downloaded from Genomics of Drug Sensitivity in Cancer (GDSC, https://www.cancerrxgene.org).

The gene sets for immune checkpoints 20, 21 and drug resistance 22 were collected in previous studies.

### Differential and chromosomal localization analysis of ATGs

Differentially expressed ATGs (|log2FoldChange| >1, false discovery rate (FDR) < 0.05) were identified by the "DESeq2" package (1.42.1) 23. The "RCircos" package (1.2.2) 24 was used for chromosomal localization.

### Functional and pathway enrichment analyses

The "clusterProfiler" package (4.10.1) was applied for gene annotation enrichment analysis 25, 26. Differential ATGs were submitted for Gene Ontology (GO) 27 and Kyoto Encyclopedia of Genes and Genomes (KEGG) 28 terms. The up- and down-regulated pathways in both ATS groups by performing gene set enrichment analysis (GSEA) 29.

### Construction and validation of ATS

The following procedure applies to all cancer types. The differential ATGs were subjected to univariate and multivariate Cox regression analysis by the "survival" package (3.5-8) 30 in each cancer type, and those with p<0.05 were retained as prognostic markers and their corresponding weights were obtained to construct ATS. The formula was as follows:



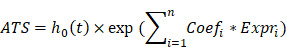



where 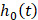 is the baseline risk function, 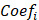 is the coefficient and 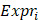 is the expression level of the ATG. The ATS was calculated for each patient according to this formula, and they were divided into high- or low-ATS groups according to the median ATS. Kaplan-Meier survival curve was performed to observe the difference of overall survival (OS) between the two groups. By comparing the clinical traits, multivariate Cox regression analysis was used to confirm the independence of the prognostic model. Finally, multiple independent external sets were used to validate.

### Assessment of correlation between ATGs and autophagy activation

The autophagy activation score was calculated using single-sample gene set enrichment analysis (ssGSEA) 31 based on a predefined gene list associated with autophagy activation 32. Subsequently, the pearson correlation between the expression of ATGs and the autophagy activation score was determined.

### Immune infiltration analysis

Multiple algorithms of immune infiltration analyses such as CIBERSORT 33, ssGSEA 31, MCPcounter 34, TIMER2 35, xCell 36, and ESTIMATE 37 were used to explore the tumor microenvironment. The pearson correlation was valued between ATS and the immune score evaluated by ESTIMATE. And the Wilcoxon rank sum test was used to compare the infiltration level of immune cell types calculated by CIBERSORT, ssGSEA, MCPcounter, xCell, and TIMER2 between the high- and low-ATS groups.

### Analysis of tumor microenvironment based on scRNA-seq data

The "Seurat" package (4.4.0) 38 was used for downstream analysis. Filtering low-quality cells was performed based on previous studies 39, 40. Data preprocessing were used the SCTransform function 41 with default settings. Uniform manifold approximation and projection (UMAP) dimensionality reduction and cell clustering were performed utilizing the top 10 calculated dimensions and a resolution of 0.5. And cell-type identification utilized the known marker genes from previous studies 39, 42. Next, autophagy-related cell clusters were identified using the Scissor approach 43. In TCGA-BLCA data, the average expression of these cell-specific markers in this cell type was used to define the score for the cell type. Cell-cell interaction was identified within high- and low-ATS samples via the "CellChat" package (1.6.1) 44.

### Association of ATS with tumor therapy response

The pearson correlation between ATS and immune checkpoint genes and drug resistance genes was calculated. Tumor immune dysfunction and exclusion (TIDE) 45 score was calculated online (http://tide.dfci.harvard.edu), and the Wilcoxon rank sum test was used to compare the TIDE scores between the high- and low-ATS groups. Based on the drug response of immunotherapy and chemotherapy data of GSE103668-BRCA and GSE67501-KIRC, respectively, receiver operating characteristic (ROC) curve and area under ROC (AUC) were performed through the "pROC" package (1.18.5) 46. Meanwhile the pearson correlation between ATS and the half-maximal inhibitory concentration (IC50) as well as the AUC value for each drug across different cancers in GDSC was calculated to select potential autophagy-related therapeutic agents (|r|>0.5, p value<0.05).

### Statistical analysis

Differences between two groups were determined by the Wilcoxon test measured. The log-rank test was applied to compare the survival statistics of categorical variables. All statistical tests were two-sided. P<0.05 was considered statistically significant. All data calculations, statistical analysis, and visualization were conducted in R 4.0.2 software.

## Results

### Pan-cancer landscape of autophagy

**Figure 1:**
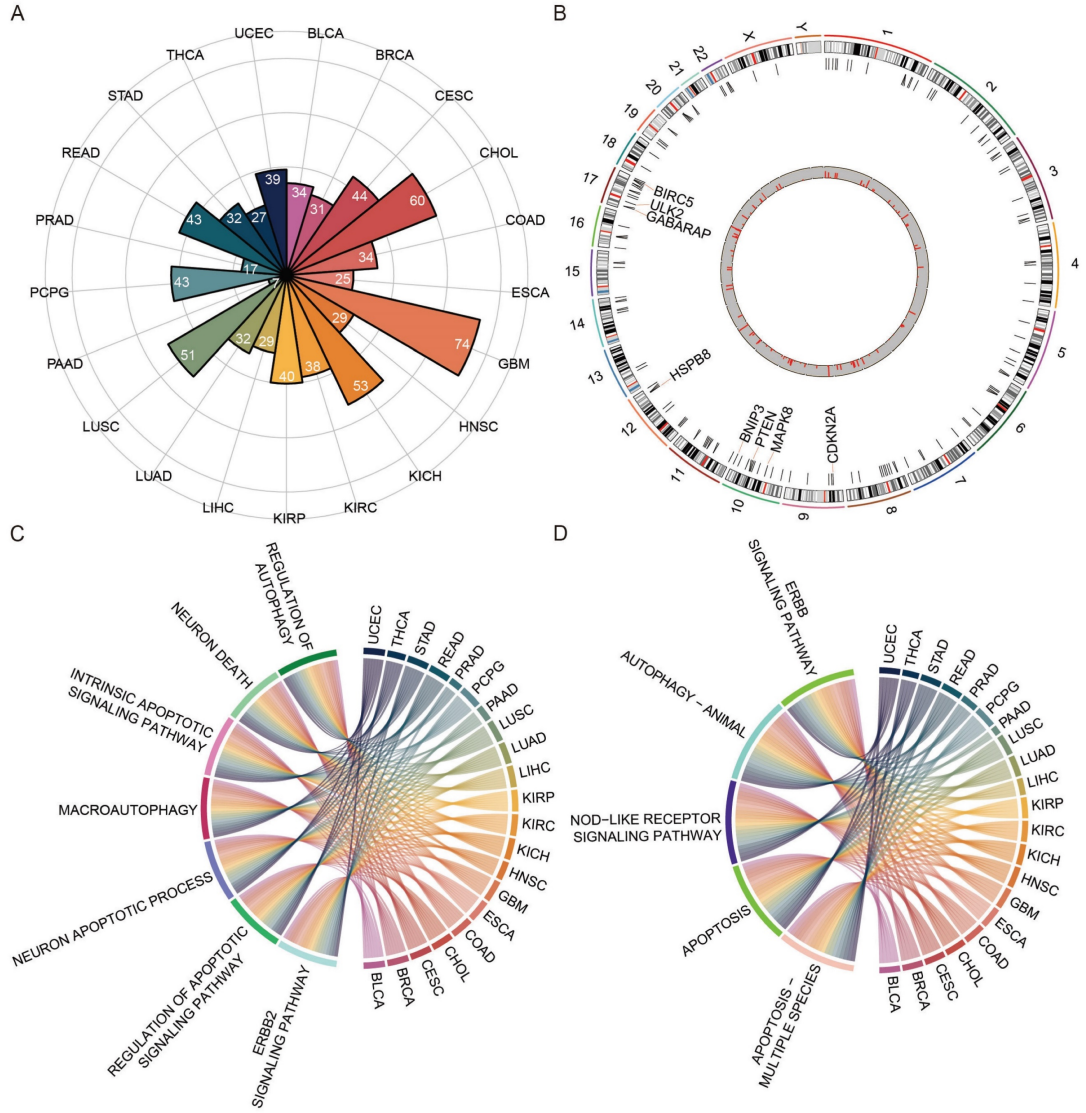
** The landscape of differential autophagy genes in pan-cancer.** (A) The number of differential ATGs in each cancer type. (B) Chromosome location and frequency of differential ATGs in cancer type. (C) The functional enrichment analyses of GO terms of ATGs in 21 cancer types. (D) KEGG pathway enrichment analysis of ATGs in 21 cancer types.

A total of 234 ATGs from HADb and a pan-cancer cohort of 8620 cases across 21 cancer types from TCGA were collected. The expression levels of ATGs were compared between cancer and matched normal samples. Approximately 3-32% of differentially expressed ATGs across 21 cancers, with the highest numbers in GBM and the lowest in PAAD (Figure 1A). The differential ATGs were distributed across 23 chromosomes, and enriched on chromosomes 10 and 17 (Figure 1B). GO and KEGG pathway enrichment analysis revealed those differential ATGs were mainly enriched in autophagy- and apoptosis-associated pathways (Figure 1C-D). Those showed that the autophagy processes in cancers were highly abnormal 47, 48.

### The ATS associated with patients' survival in 11 cancers

**Figure 2:**
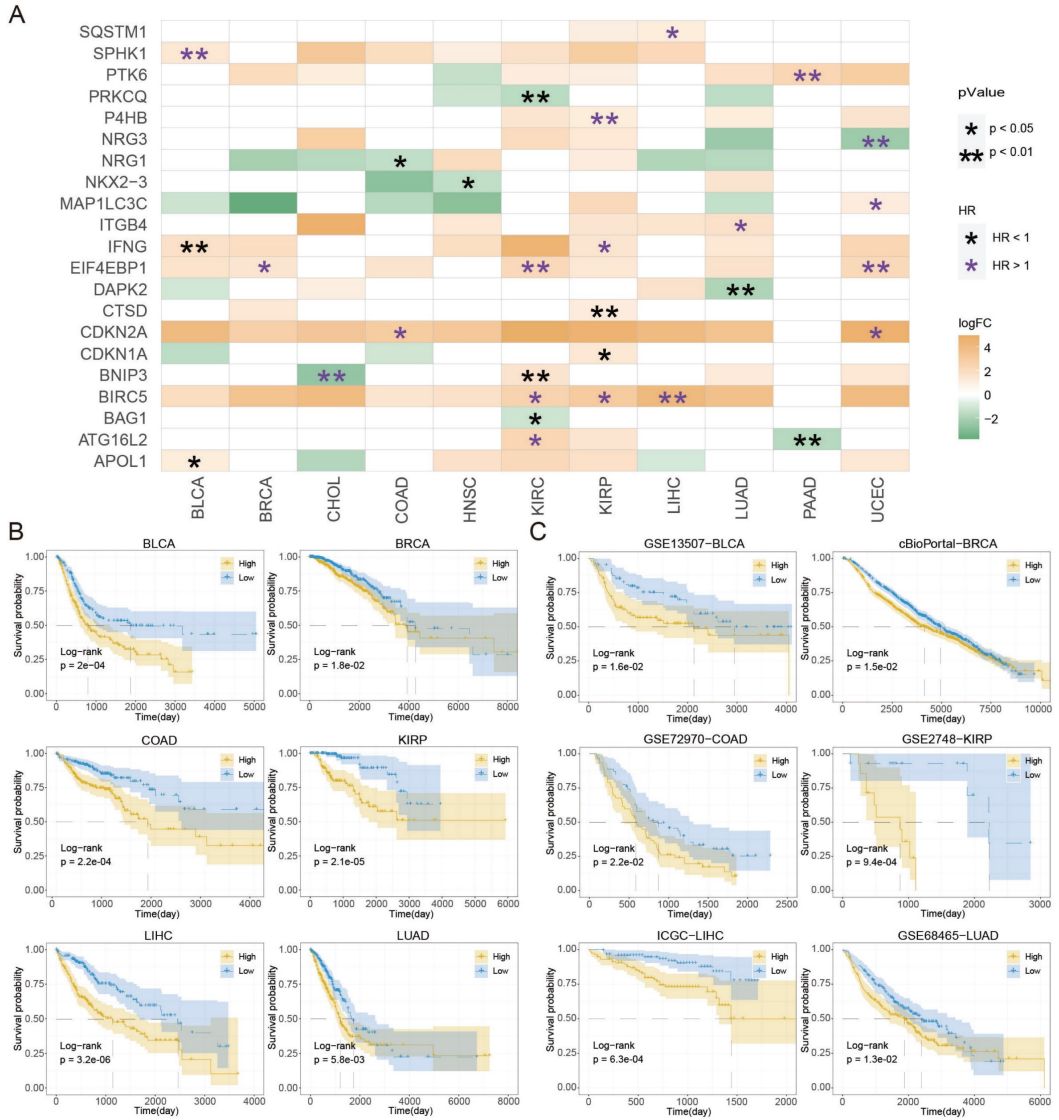
**Prognostic roles of autophagy in pan-cancer.** (A) The differential ATGs with clinical relevance in each cancer type. (B) Kaplan-Meier curves of OS of low and high groups stratified by the ATS in six cancer types (BLCA, BRCA, COAD, KIRP, LIHC, and LUAD) in training data. (C) Likewise, GSE13507-BLCA, cBioPortal-BRCA, GSE72970-COAD, GSE2748-KIRP, ICGC-LIHC, and GSE68465-LUAD patients in validation data.

A total of 55 prognosis-related ATGs of 11 cancer types were identified through univariate Cox regression analysis respectively (Table S1). Subsequently, a multivariate Cox regression model was used to select feature ATGs for each cancer, and 21 ATGs were left for 11 cancer types (Table S2). The ATS was calculated for each cancer using its specific set of ATGs. Among those genes, *BIRC5* and *EIF4EBP1* exhibited higher expression and were associated with poor clinical outcomes in KIRC, KIRP, and LIHC; *IFNG* had elevated expression and different clinical outcomes in BLCA and KIRP; *ATG16L2* and *BNIP3* showed various expression and were associated with clinical outcomes in multiple cancer types (Figure 2A). In addition, the relationship between the extent of autophagy activation and the expression of ATGs identified through Cox regression analysis was examined (Figure S1). In BLCA, *SPHK1* showed a significant correlation (cor=0.325, P=1.293e-11); *BIRC5* was correlated in both LIHC (cor=-0.482, P=5.219e-23) and KIRP (cor=-0.44, P=4.608e-15); *BAG1* was significantly correlated in KIRC (cor=0.376, P=1.807e-19). These findings suggest a connection between ATGs and tumor autophagy. These results demonstrated that autophagy might assist in predicting prognosis.

**Figure 3:**
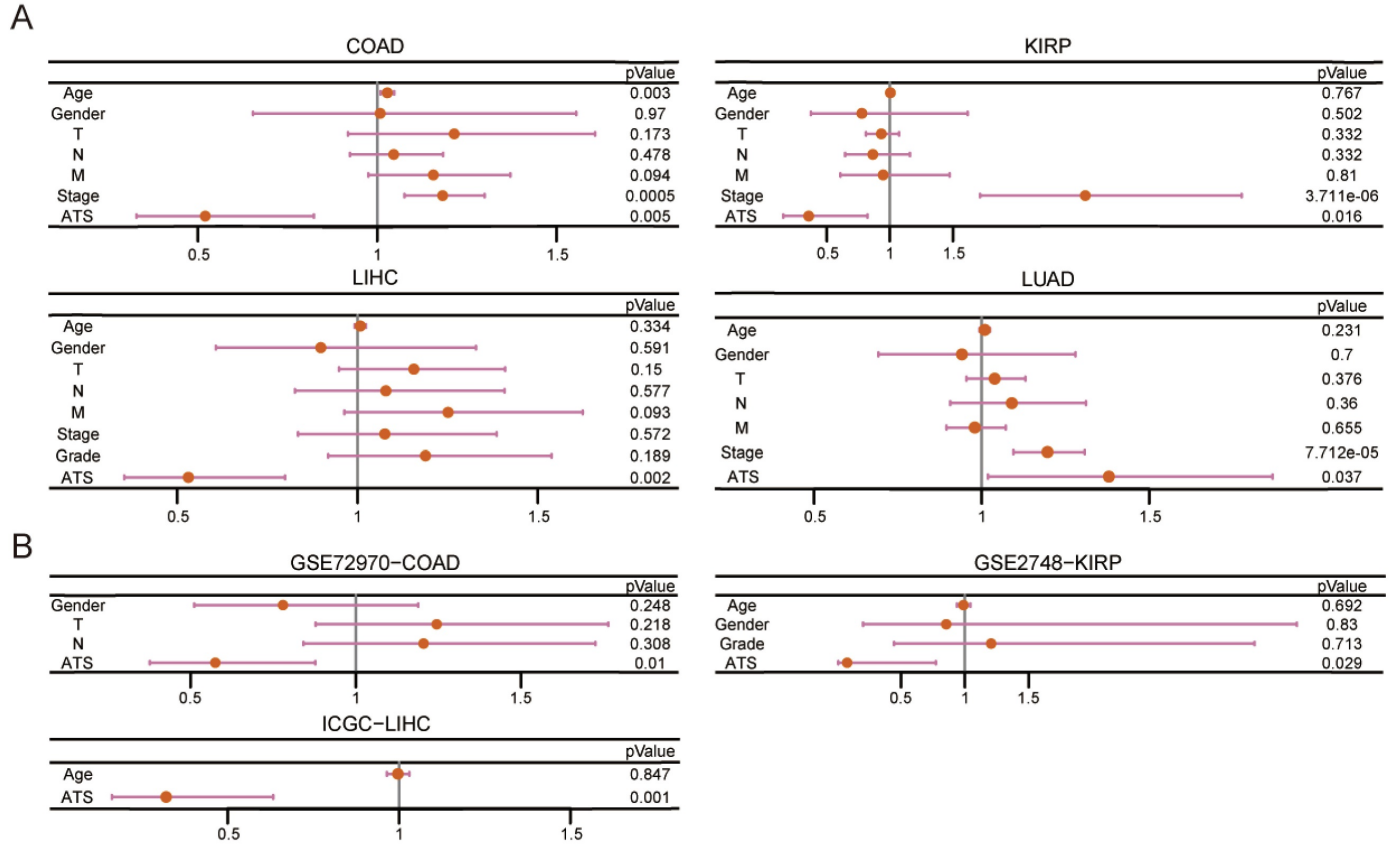
** The ATS is an independent prognostic factor.** (A) Multivariate Cox regression of prognosis factor for OS of LIHC, COAD, KIRP, and LUAD patients in training data. (B) Likewise, GSE72970-COAD, GSE2748-KIRP, and ICGC-LIHC patients in validation data.

Kaplan-Meier plot revealed that patients in the high-ATS group had a poorer prognosis than those in the low-ATS group in 11 cancers (Figure 2B and Figure S2A). To validate the predictive value of the ATS for OS, six validation Cohorts from GEO, ICGC, and cBioPortal were analyzed. The high ATS was still correlated with poor OS (Figure 2C). ATS was an independent prognostic factor for patients of CHOL (HR=5.537, 95% CI= 1.639~18.706; P=0.006), COAD (HR=1.922, 95% CI=1.215~3.04; P=0.005), KIRC (HR=1.997, 95% CI=1.401~2.846; P=1.291e-04), KIRP (HR=2.796, 95% CI=1.216~6.428; P=0.016), LIHC (HR=1.883, 95% CI=1.251~2.833; P=0.002), LUAD (HR=1.38, 95% CI=1.019~1.869; P=0.037), PAAD (HR=2.004, 95% CI=1.287~3.119; P=0.002) and UCEC (HR=2.448, 95% CI=1.462~4.101; P=6.689e-04) through multivariate Cox regression analysis (Figure 3A and Figure S2B). In validation cohorts (GSE72970-COAD, GSE2748-KIRP, and ICGC-LIHC), the results were consistent with the TCGA training sets (Figure 3B). These results indicated that the ATS could be a robust indicator for prognosis in various cancers.

### The ATS correlated with tumor immunity

**Figure 4:**
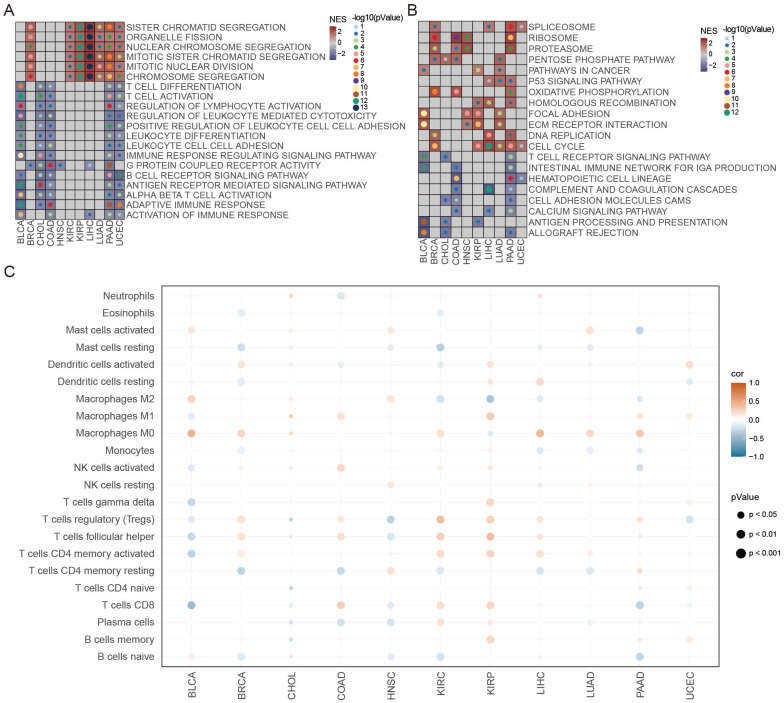
**The functional enrichment and tumor immune microenvironment analyses of ATS.** (A-B) GSEA analysis of differentially expressed genes between the high- and low-autophagy groups in each cancer. (A) GO. (B) KEGG. (C) Correlation between ATS and immune cells estimated by the CIBERSORT algorithm in each cancer.

The biological features of differential genes between the high- and low-ATS groups were clarified using GSEA. As revealed by Figure 4A-B, the high-ATS groups were mainly involved in cell division and energy metabolism processes such as chromosome segregation, organelle fission, and oxidative phosphorylation, indicating that autophagy was related to cell cycle progression; the low-ATS groups concentrated on T cell activation and differentiation, regulation of lymphocyte activation, antigen processing and presentation, and B cell receptor signaling pathway and other immune-related processes, suggesting that autophagy was associated with the tumor immune microenvironment. Moreover, the correlation between ATS and the abundance of immune cell infiltration evaluated by the CIBERSORT algorithm was examined. The ATS was significantly negatively correlated with infiltration abundance of CD8 T cells and M1 macrophages in BLCA, but positively correlated in COAD and KIRP (Figure 4C). This implies that autophagy might contribute to inhibiting or stimulating immune responses. In addition, the ATS was also correlated with infiltration abundance of activated dendritic cells, Tregs, and activated NK cells (Figure 4C). We also observed that the ATS was significantly negatively correlated with immune scores calculated by the ESTIMATE algorithm in BLCA, but positively correlated in KIRC (Figure S3A-B). The infiltration abundance of CD8 T cells evaluated by the ssGSEA, MCPcounter, and xCell algorithm was higher in the low-ATS group in BLCA (Figure S3C), while lower in KIRC (Figure S3D). These results were consistent with those calculated using CIBERSORT.

### Association between autophagy and tumor microenvironment in BLCA and KIRC based on scRNA-seq data

**Figure 5:**
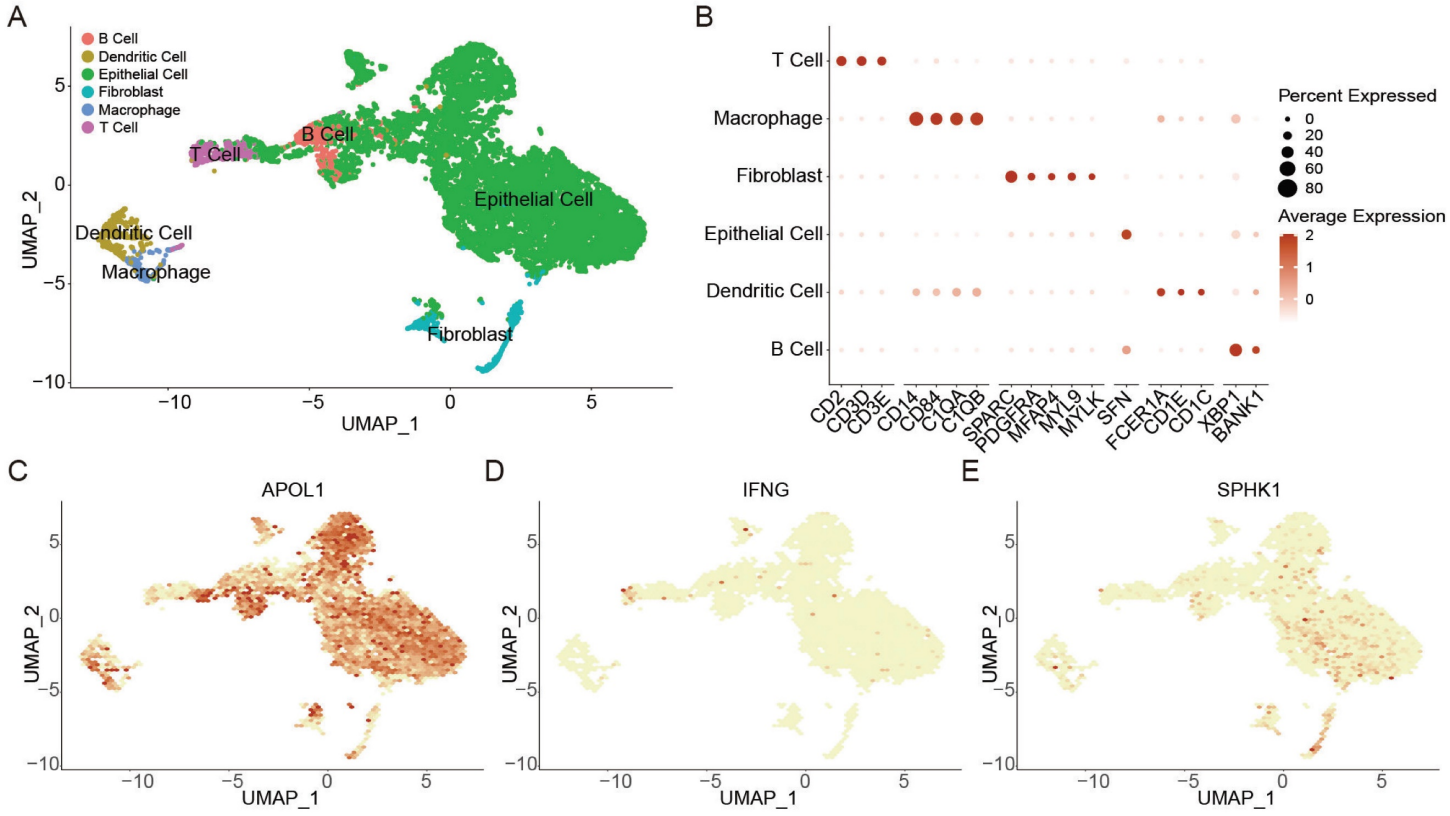
**The effect of autophagy on the tumor microenvironment at the single-cell level in BLCA.** (A) UMAP plot showing the annotation and color codes for cell types in the BLCA ecosystem. (B) Bubble plot showing the expression levels of marker genes in each cell type. (C-E) UMAP visualized plot showing the expression of genes for ATS of BLCA. (C) *APOL1*. (D) *IFNG*. (E) *SPHK1*.

A total of 17,384 cells in 4 BLCA patients from two scRNA-seq datasets (GSE190888 and GSE186520) were used to dissect the mechanisms of autophagy associated with immune microenvironment. Using canonical cell-type markers (Figure 5B), these cells were identified into 6 cell types (Figure 5A). Non-immune cells primarily consisted of epithelial cells and fibroblasts. The identified immune cells included B cells, dendritic cells, macrophages, and T cells (Figure 5A). Next, the expression of ATS genes (*APOL1*, *IFNG*, and *SPHK1*) in BLCA were observed. High expression of *APOL1* in the tumor microenvironment indicated that it played an important role in tumor invasion and immune defense 49 (Figure 5C); T cells displayed a high expression level of *IFNG*, mediating T cell killing to suppress tumors in immune regulation 50 (Figure 5D); *SPHK1* was highly expressed in epithelial cells and fibroblasts, which might regulate fibroblast differentiation to promote cancer proliferation 51-54 (Figure 5E).

**Figure 6:**
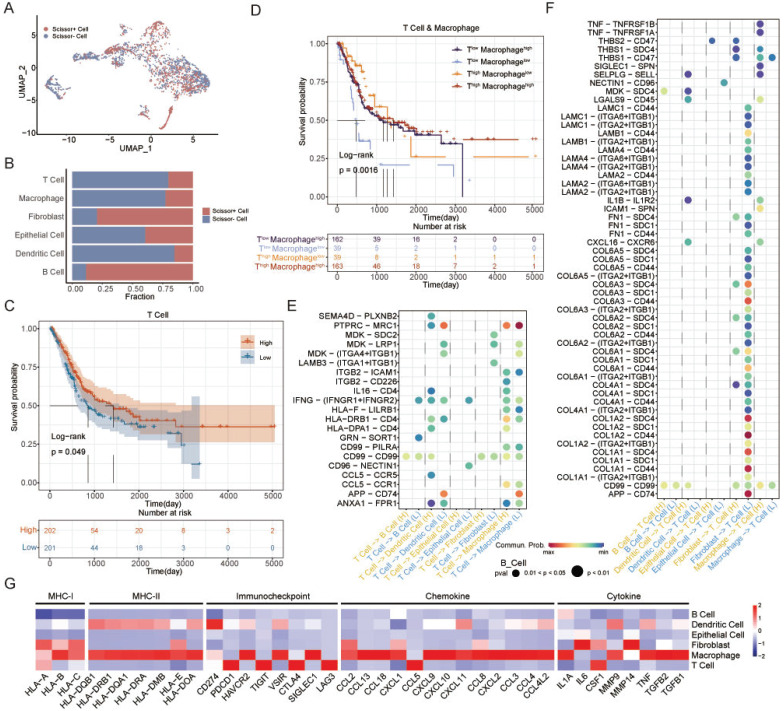
**Identification of autophagy-related T cells and differences of T-cell interactions associated with different ATS in BLCA.** (A) The UMAP visualization of the Scissor-method selected cells. The red and blue dots are cells associated with the autophagy phenotype. (B) Bar plot showing cell proportion in each cell type by Scissor results. (C) Survival analysis of OS between the high and low T cell expression in TCGA-BLCA. (D) Survival analysis of OS between the T^high^ Macrophage^high^, T^high^ Macrophage^low^, T^low^ Macrophage^high^, and T^low^ Macrophage^low^ in TCGA-BLCA. (E-F) Comparison of the ligand-receptor pairs in the cell-cell communication between the high- and low-ATS groups. Bubble chart showing the interaction between (E) T cells and other cells, (F) other cells and T cells, based on selected ligand and receptor pairs. (G) Heatmap displaying the expression levels of selected immunity/inflammatory genes across six cell types.

The Scissor algorithm can identify cell subgroups that are most relevant to a given phenotype by quantifying the similarity between each single cell and each bulk sample in the scRNA-seq data 43. ATS-related cells were identified and classified into Scissor+ and Scissor- cell subgroups using the Scissor algorithm (Figure 6A). The Scissor- group was negatively associated with the ATS, and accounted for the majority of T cells, macrophages, and dendritic cells, and a small proportion of fibroblasts and B cells (Figure 6B). The T-cell and macrophage expression scores of TCGA-BLCA samples were calculated based on the T-cell and macrophage markers, respectively (Figure S4A-B). The samples were stratified into high or low expression groups according to the median value. Kaplan-Meier analysis revealed that the T-cell^high^ group was correlated with better clinical prognosis (P=0.049; Figure 6C), while macrophage was not associated with patient survival (P=0.46; Figure S4C). However, patients with Macrophage^high^ had the better OS than Macrophage^low^ in T-cell^low^ group (P=0.0016; Figure 6D). This suggested that interactions between T cells and macrophages could regulate immune killing mechanisms and improve patient outcomes. Next, to investigate the interaction network of T cells in high- and low-ATS groups, potential ligand-receptor pairs were calculated using CellChat.

Notably, in MK signaling pathway, ligand *MDK* and its multiple receptors, such as *SDC2*, *LRP1*, and *ITGA4*/*ITGB1*, were only active in the low-ATS group from T cells to dendritic cells, fibroblasts, and macrophages; in IFN-II signaling pathway, *IFNG* with multi-subunit receptor *IFNGR1*/*IFNGR2* was active in the low-ATS group from T cells to B cells and epithelial cells and was more active in the low-ATS group from T cells to dendritic cells and macrophages than in the high-ATS group (Figure 6E). Multiple ligand-receptor pairs from ECM-receptor interaction pathways were found to be highly active in the low-ATS group from fibroblasts to T cells; ligand *TNF* with receptors *TNFRSF1A* and *TNFRSF1B* was active in the high-ATS group from macrophages to T cells (Figure 6F). Those interactions between T cells and other cells in low- and high-ATS groups might activate and regulate T cell function to exert effects on the tumor microenvironment, which led to survival differences. These findings suggest that interactions between T cells and macrophages contribute to improve patient survival. Additionally, the biological functions of the selected genes were described (Figure 6G). For example, both macrophages and T cells exhibited high expression levels of inflammatory/immune-related genes. It was also observed that these cells expressed elevated levels of immune checkpoint molecules, for example, macrophages had high levels of *HAVCR2*, *VSIR*, and *SIGLEC1*, and T cells showed high levels of *PDCD1*, *TIGIT*, *CTLA4*, and *LAG3*.

Besides, we explored the relationship between autophagy and the tumor microenvironment in KIRC. Totals of 12,239 cells in three KIRC patients in the GSE121638 dataset were clustered into six cell types, including B cells, DCs, macrophages, monocytes, NK cells, and T cells (Figure S5A). We observed that *ATG16L2* was highly expressed in NK cells and T cells, potentially regulating their activation 55 (Figure S5B). Additionally, the high expression of BIRC5 in macrophages suggests its important role in tumor immune modulation 56 (Figure S5C). Using CellChat, we constructed an interaction network, and found that *IFNG*, interacting with its multi-subunit receptors *IFNGR1/IFNGR2*, was more active in the high-ATS group from T cells to DCs, macrophages, and monocytes compared to the low-ATS group; multiple ligand-receptor pairs involved in ECM-receptor interaction pathways were highly active in the high-ATS group from monocytes to T cells (Figure S5D-E). Meanwhile, macrophages, monocytes, NK cells, and T cells exhibited high expression levels of inflammatory/immune-related genes, indicating their potent antitumor effects (Figure S5F). These results demonstrate that autophagy influences tumor immunity through diverse mechanisms.

### The ATS associated with cancer treatment

**Figure 7:**
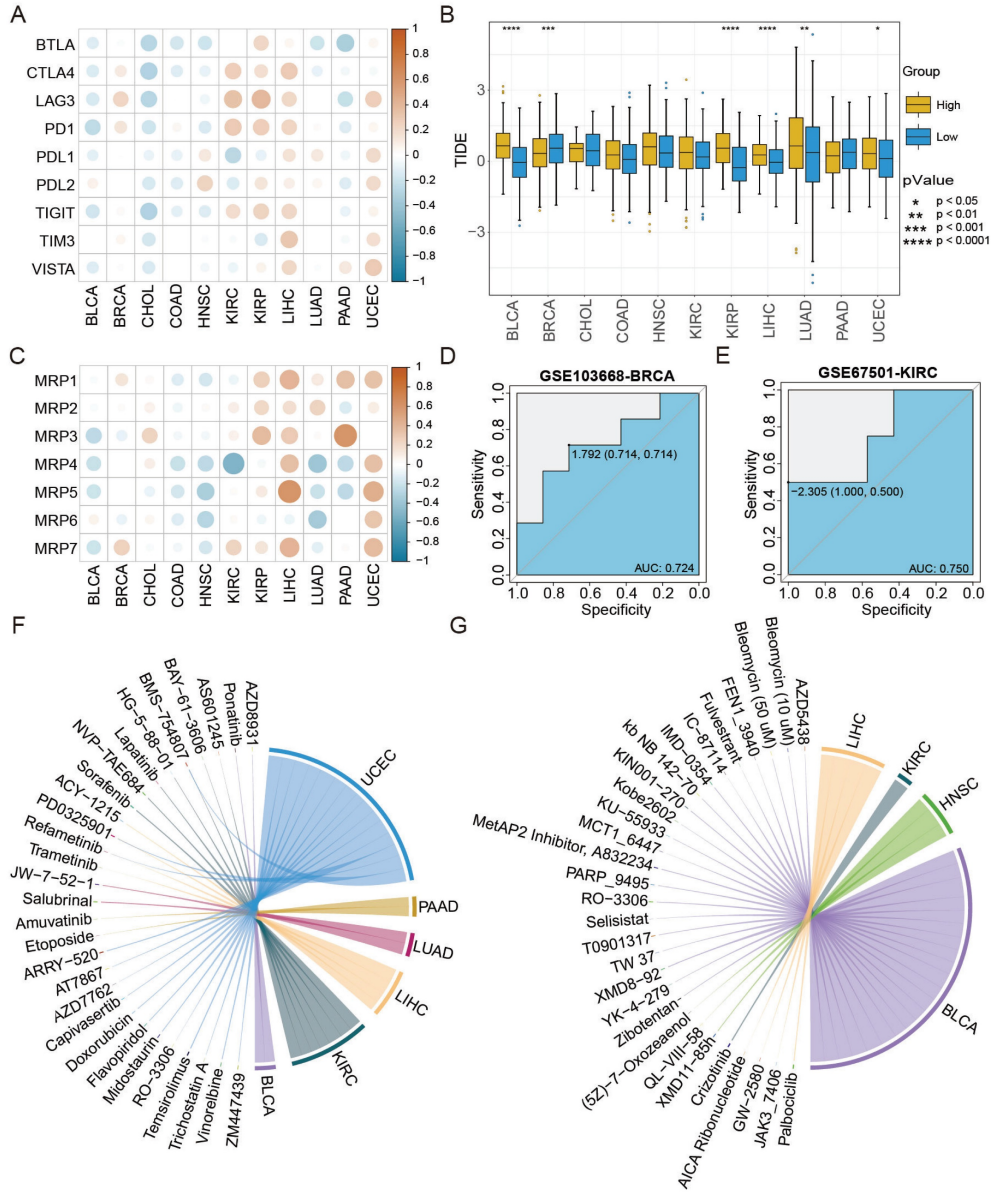
**ATS shows stronger predictive ability for immunotherapy and chemotherapy response.** (A) Pearson correlation between ATS and expression levels of immune checkpoint genes. (B) Differences in TIDE scores between the high- and low-ATS groups. (C) Pearson correlation between ATS and expression levels of drug resistance genes. (D-E) Receiver operating characteristic curves of ATS to predict chemotherapy and immunotherapy response in (D) GSE103668-BRCA with chemotherapy cohort and (E) GSE67501-KIRC with immunotherapy cohort. (F-G) Pearson correlation between ATS and the IC50 value. (F) Positive correlation. (G) Negative correlation.

The ATS was associated with therapy response based on analyses of immune checkpoints, TIDE scores, and drug resistance genes. First, the ATS was negatively correlated with expression levels of most immune checkpoint genes in BLCA and CHOL, but positively correlated in BRCA, LIHC, and UCEC (Figure 7A). Next, the distribution of TIDE scores was compared between the high- and low-ATS groups. As shown in Figure 7B, TIDE scores were lower in the low-ATS group in BLCA (P=6.7e-15), KIRP (P=4.7e-09), and LIHC (P=5.1e-05), while higher in BRCA (P=7.3e-04). Last, the ATS was found to be negatively correlated with expression levels of drug resistance genes in BLCA and HNSC, while positively correlated in KIRP, LIHC, and UCEC (Figure 7C).

Furthermore, the AUC value was used to evaluate the accuracy of ATS in predicting treatment response. The therapy response achieved AUC of 0.724 and 0.75 for GSE103668-BRCA and GSE67501-KIRC, respectively (Figure 7D-E), suggesting the potential of the ATS as an indicator of therapy response. Besides, the correlation between the ATS and both IC50 and AUC values was calculated. The analysis revealed that the ATS was positively correlated with the IC50 (cor=0.527, P=2.056e-02) and AUC (cor=0.59, P=7.832e-03) of ponatinib in BLCA, but negatively correlated with IC50 (cor=-0.647, P=4.991e-03) and AUC (cor=-0.647, P=5.034e-03) of GW2580 in LIHC as well as with the IC50 (cor=-0.664, P=3.649e-03) and AUC (cor=-0.536, P=2.641e-02) of fulvestrant in BLCA (Figure 7F-G, Figure S6, Table S3, Table S4). Taken together, ATS might serve as an indicator for the response to different anti-cancer treatments.

## Discussion

Differentially expressed ATGs on the chromosomes were mainly enriched on chromosomes 10 and 17, such as *BIRC5*, *BNIP3* and *SQSTM1*. Accumulating evidence has suggested those chromosomes harbor some key genes that influence cancer development and tumor prognosis. For instance, high *BIRC5* expression may promote the proliferation of LIHC and reduce their susceptibility to chemoradiotherapy, leading to poor prognosis 57. *BNIP3* induces autophagy to degrade protein aggregates and dysfunctional organelles that can be harmful to the cell 58. At the same time, arsenic trioxide induces autophagic cell death in malignant glioma cells by upregulation of BNIP3 59; loss of *BNIP3* expression results in a more aggressive tumor phenotype correlating with worsened prognosis in patients with pancreatic cancer and increases resistance to 5-fluoro-uracil and gemcitabine 60. *SQSTM1* regulates metabolism through autophagy to provide nutrients that favor the survival and proliferation of tumor cells. *SQSTM1* also impairs the presentation by antigen-presenting cells through autophagy failing to generate an effective anti-tumor cytotoxic T-cell response, which allows tumor cells to escape immune surveillance, which can promote LIHC progression and produce a poor survival rate 61, 62.

Enrichment analysis showed that the high-ATS group was mainly related to signaling pathways that control cell proliferation, and the low-ATS group was primarily concerned with the immune-inflammatory signaling pathways. Further exploration of the relationship between autophagy and tumor immunity found that autophagy could inhibit cancer progression by regulating the immune microenvironment or promote cancer proliferation in the exact opposite way 1, 3, 7, 63. High CD8T cell and M1 macrophage infiltration were observed in the BLCA low-ATS group, which may be the reason why low-ATS patients have a better prognosis than high-ATS patients in BLCA. Interestingly, in the KIRP high-ATS group, patients had high CD8T cell infiltration but poor prognosis. It might be that exhausted CD8 T cells positively correlated with ATS (R=0.23, P=7.8e-05), resulting in the inability of CD8 T cells to exert their antitumor effects.

Next, BLCA was taken as an example to describe in detail the impact of autophagy-regulated tumor microenvironment on patient prognosis based on scRNA-seq data. It was found that *IFNG*-(*IFNGR1*+*IFNGR2*) was more active in the low-ATS group from T cells to B cells, DCs, and macrophages, which could regulate macrophages and DCs antigen presentation, B-cell proliferation and differentiation, and T-cell recruitment 64, 65. Meanwhile, *TNF*-*TNFRSF1A*, *TNF*-*TNFRSF1B*, and *IL1B*-*IL1R2* were found to activate in the high-ATS group from macrophages to T cells, which exerted anti-inflammatory effects to suppress immunity 66-68. That was why interactions between T cells and macrophages in the low-ATS group could result in a good prognosis. Those showed that autophagy induced different immune cell interactions and affected the prognosis of patients, providing novel insights into the tumor microenvironment.

Last, the relationship between ATS and therapy response was analyzed to explore the practical value of ATS. Most of the cancer patients in the low-ATS group had a lower level of TIDE score, which indicated that they were more inclined to immunotherapy. Besides, a variety of drugs were related to ATS in different cancers, providing a novel guide to drug repositioning (Figure 7F-G). For example, chloroquine or hydroxychloroquine enhances the cytotoxicity of a few drugs, such as ponatinib 69, 70, by inhibiting autophagy to improve the antitumor effect on cancer cells; cellular sensitivity to fulvestrant can be enhanced by increasing autophagic activity 71. GW2580 treatment significantly increased the proportion of M1 macrophages by inhibiting autophagy to improve cancer outcome 72, 73. Therefore, the ATS can also be used as a potential indicator of clinical treatment.

Collectively, the potential implications of autophagy in prognosis and therapy response across multiple cancer types were elucidated in this study. The ATGs drived diverse regulatory mechanisms in the tumor microenvironment leading to distinct clinical outcomes. Further, ATS can serve as a reference for predicting patient prognosis and treatment response. Our findings will provide a recommendation for precision medicine in cancers.

## Supplementary Material

Supplementary figures and tables.
