# Supplementary material for: Pan-cancer analyses reveal cancer-type-specific autophagy signatures with potential implications for prognosis and therapy response
Source: J Cancer. 2025 Jan 1;16(1):122–34. doi: 10.7150/jca.101046 (PMC11660141; doi:10.7150/jca.101046)
Supplement: Supplementary file 1 — Supplementary figures and tables. [file jcav16p0122s1.pdf]

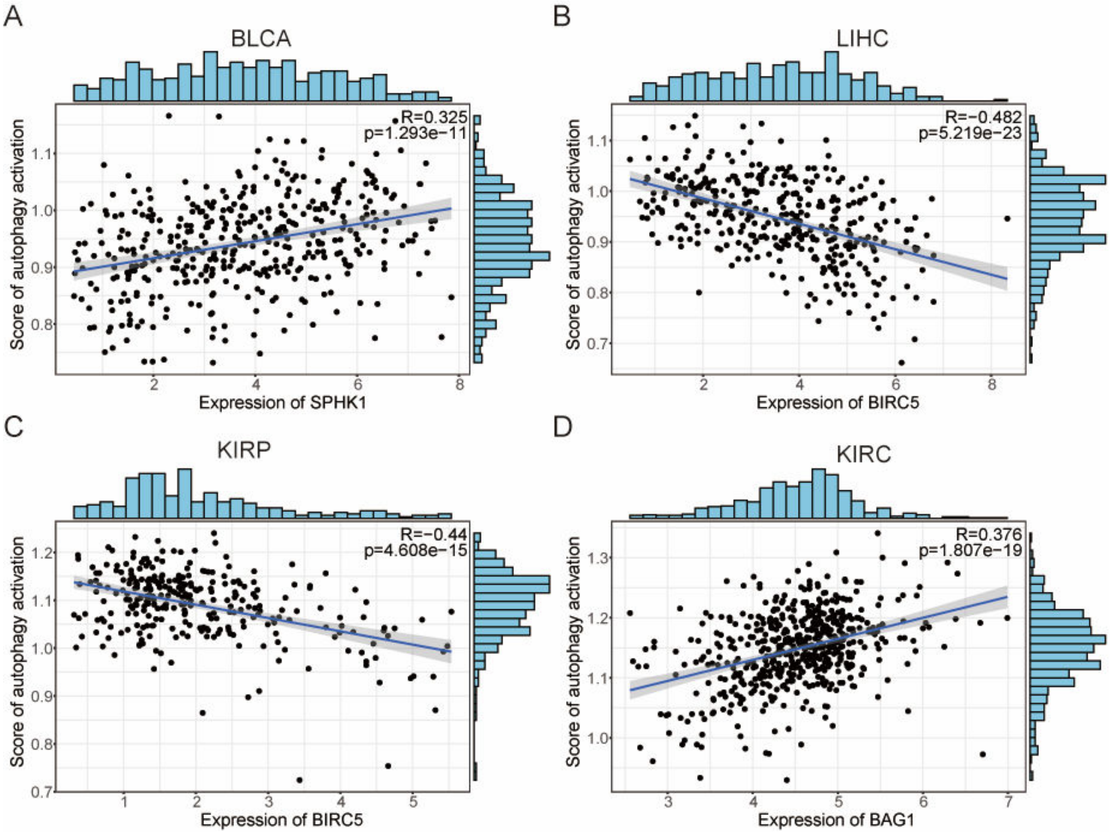

Figure S1. Correlation between the ATGs and the score of autophagy activation. (A) *SPHK1* in BLCA. (B) *BIRC5* in LIHC. (C) *BIRC5* in KIRP. (D) *BAG1* in KIRC.

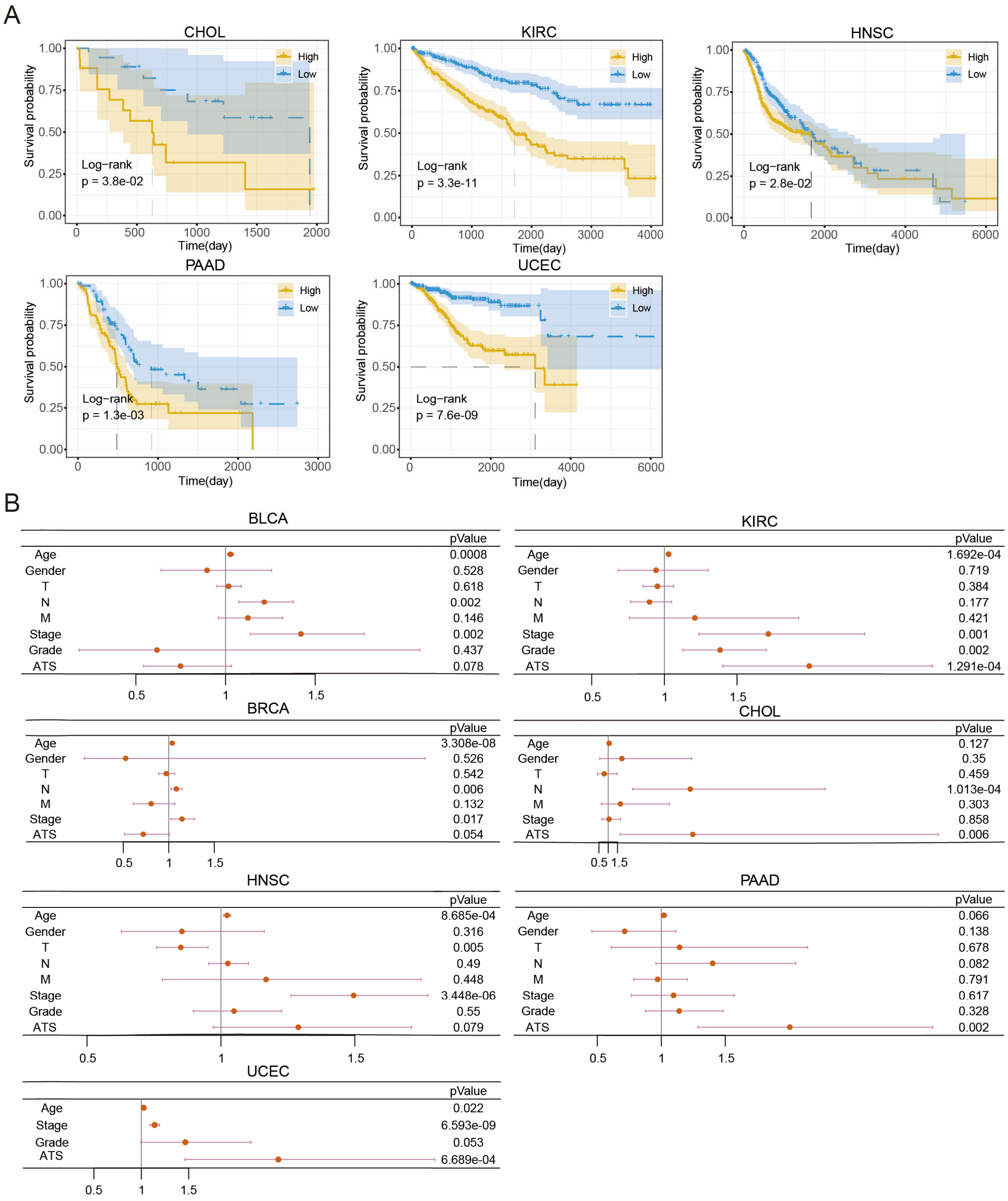

Figure S2. Prognosis analysis of ATS. (A) Survival analysis of ATS in CHOL, KIRC, HNSC, PAAD, and UCEC. (B) Multivariate Cox regression analysis of ATS in BLCA, BRCA, CHOL, KIRC, HNSC, PAAD, and UCEC.

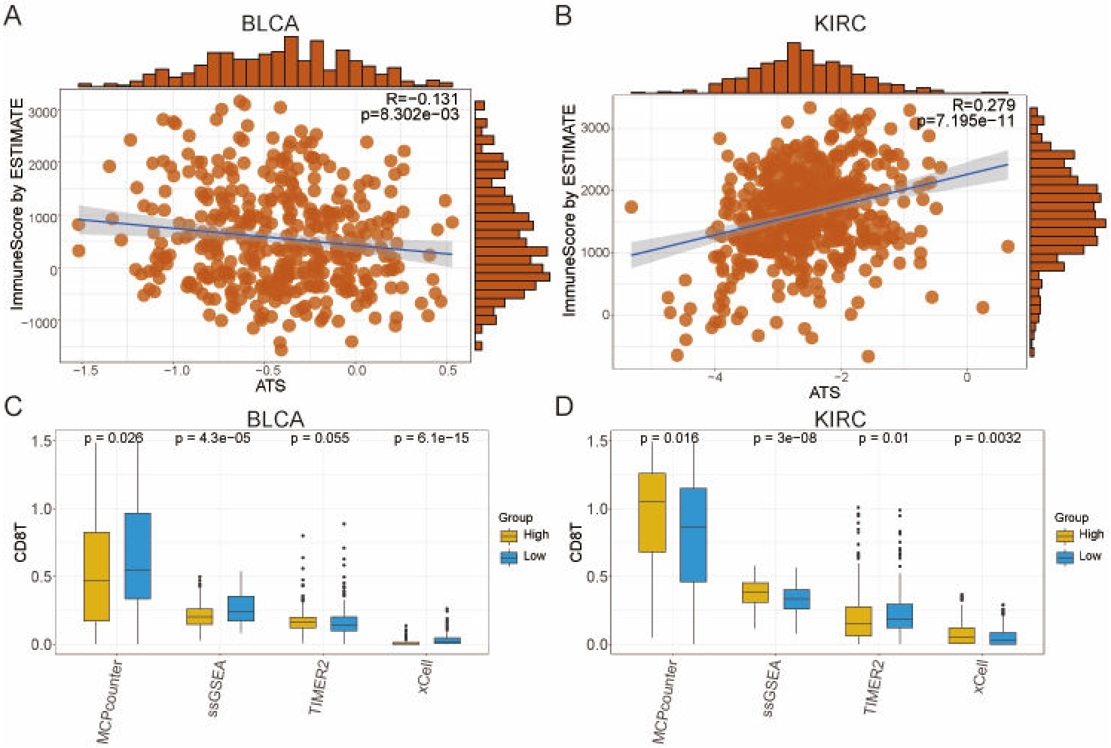

Figure S3. Relationship between the ATS and tumor immune by ssGSEA, MCPcounter, TIMER2, xCell and ESTIMATE. (A-B) Correlation between the ATS and immune scores in (A) BLCA and (B) KIRC. (C-D) Differences in CD8 T cell infiltration between the high- and low-ATS groups in (C) BLCA and (D) KIRC.

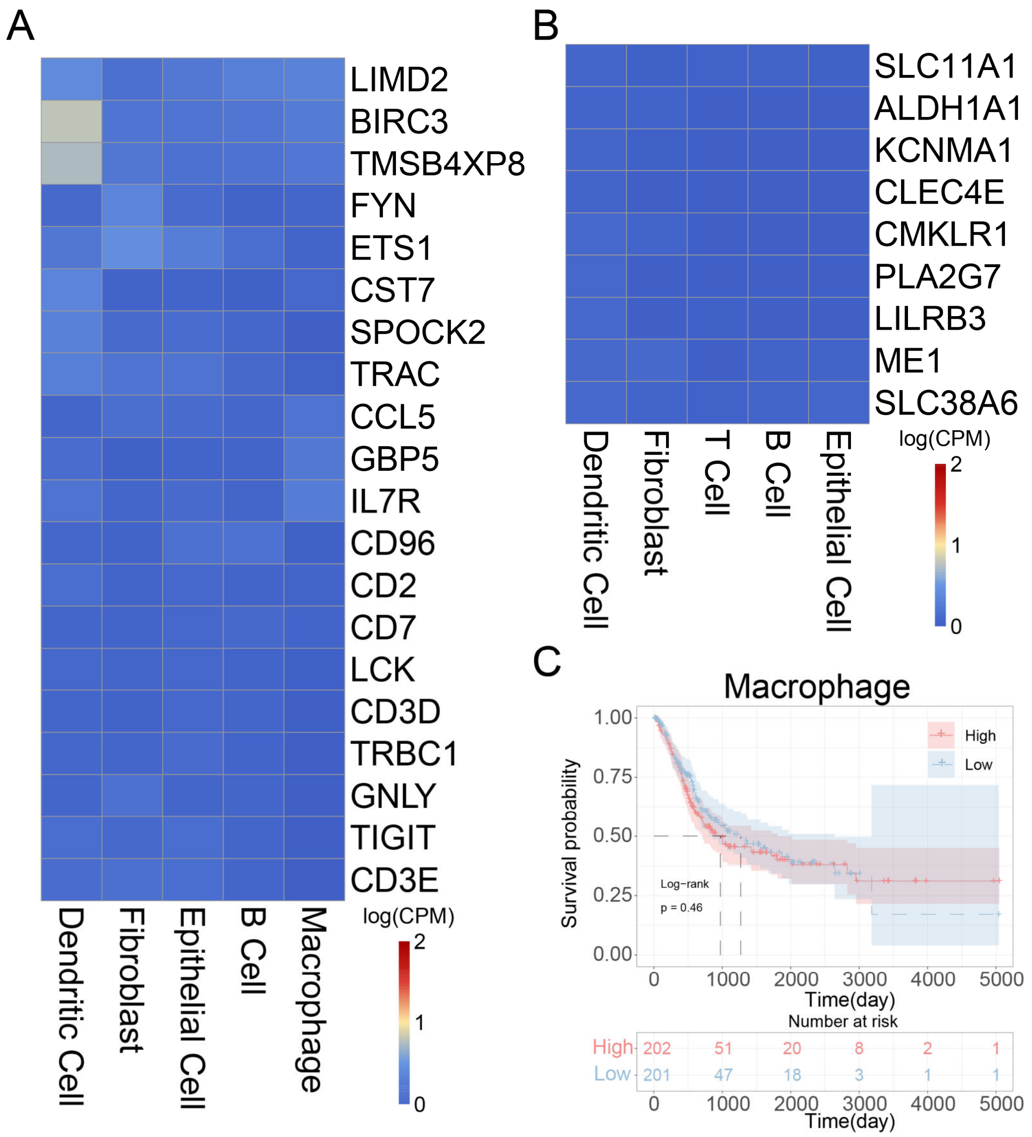

Figure S4. Assessment of T cell and macrophage signatures in single cell and bulk data. (A) Expression of putative signature genes for T cells in non-T cell scRNA-seq compartments from GSE103668 and GSE67501 datasets. (B) Expression of putative signature genes for macrophage cells in non-macrophage scRNA-seq compartments from GSE103668 and GSE67501 datasets. (C) Survival analysis of OS between the high and low macrophage expression in TCGA-BLCA.

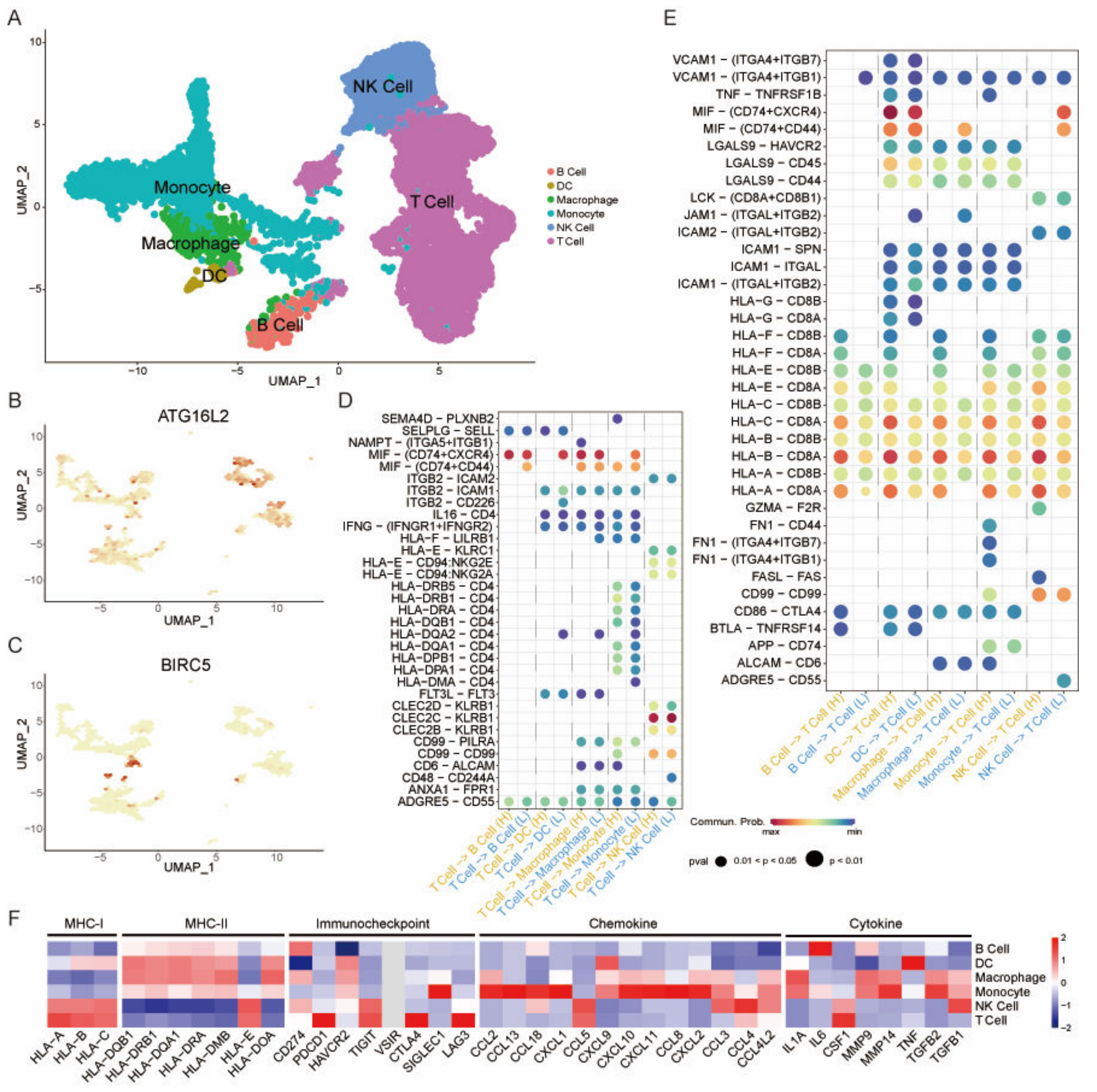

Figure S5. Impact of autophagy on the tumor microenvironment at the single-cell level in KIRC.

(A) UMAP plot showing cell type annotations and corresponding color codes within the KIRC ecosystem. (B-C) UMAP visualized plot showing the expression of genes for ATS of KIRC. (B) *ATG16L2*. (C) *BIRC5*. (D-E) Comparison of the ligand-receptor pairs in the cell-cell communication between the high- and low-ATS groups. Bubble chart showing the interaction between (D) T cells and other cells, (E) other cells and T cells, based on selected ligand and receptor pairs. (F) Heatmap showing the expression levels of immunity/inflammatory genes across six cell types.

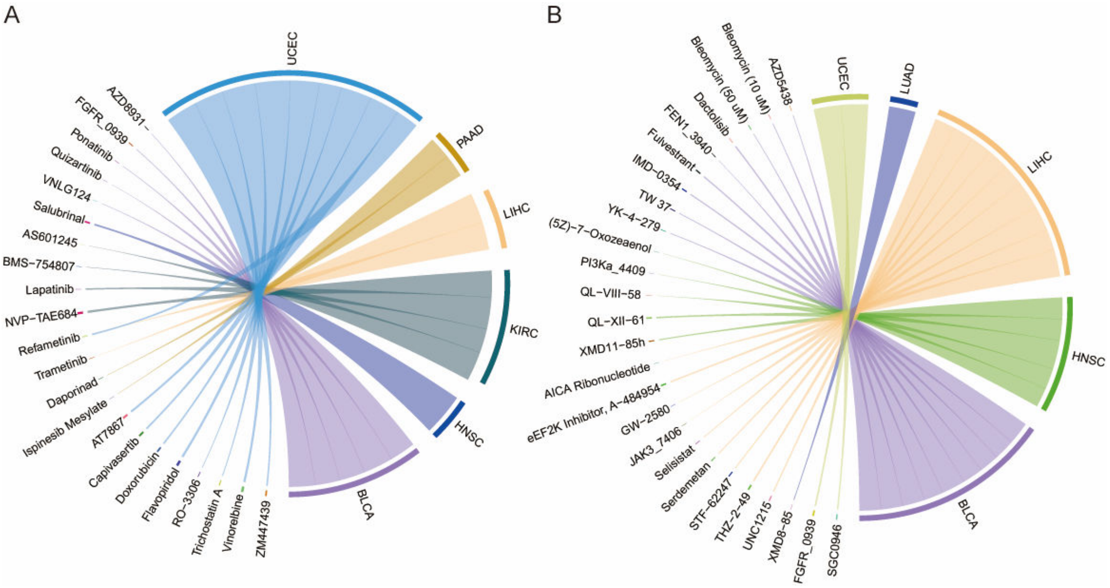

Figure S6. Correlation between ATS and the AUC value. (A) Positive correlation. (B) Negative correlation.

Table S1 Univariate Cox regression analysis of differential ATGs for OS across 11 cancer types in training data.

Table S1-1 Univariate Cox regression analysis of differential ATGs for OS across BLCA in training data.

gene	P Value	HR (95%CI)
BID	0.006	0.768 (0.636-0.927)
IFNG	0.018	0.927 (0.870-0.987)
APOL1	1.115e-05	0.822 (0.753-0.897)
SPHK1	0.028	1.094 (1.010-1.186)
DIRAS3	0.024	1.107 (1.013-1.209)
ITGA3	0.008	0.886 (0.810-0.969)

Table S1-2 Univariate Cox regression analysis of differential ATGs for OS across BRCA in training data.

gene	P Value	HR (95%CI)
JUN	0.046	0.856 (0.736-0.997)
PTK6	0.028	1.139 (1.014-1.281)
TP63	0.004	0.902 (0.841-0.967)
IFNG	0.010	0.900 (0.830-0.975)
PPP1R15A	0.046	0.822 (0.677-0.997)
NRG1	0.048	0.925 (0.856-0.999)
NRG2	0.042	0.910 (0.831-0.997)
EIF4EBP1	0.008	1.196 (1.047-1.366)

Table S1-3 Univariate Cox regression analysis of differential ATGs for OS across CHOL in training data.

gene	P Value	HR (95%CI)
JUN	0.013	0.457 (0.245-0.849)
EGFR	0.040	0.716 (0.521-0.984)
CAPN1	0.050	0.483 (0.233-1.000)
BNIP3	0.016	1.912 (1.131-3.233)
BCL2	0.050	0.743 (0.552-1.000)

Table S1-4 Univariate Cox regression analysis of differential ATGs for OS across COAD in training data.

gene	P Value	HR (95%CI)
SPHK1	0.049	1.144 (1.001-1.308)
NRG1	0.010	0.863 (0.772-0.965)
CDKN2A	0.004	1.182 (1.056-1.323)

Table S1-5 Univariate Cox regression analysis of differential ATGs for OS across HNSC in training data.

gene	P Value	HR (95%CI)
RAB32	0.003	1.282 (1.087-1.514)
EGFR	0.017	1.124 (1.021-1.238)
PINK1	0.026	1.213 (1.024-1.436)
NKX2-3	0.009	0.936 (0.890-0.984)
DDIT3	0.046	1.131 (1.002-1.276)
FADD	6.672e-05	1.229 (1.110-1.360)

Continued Table S1-5 Univariate Cox regression analysis of differential ATGs for OS across HNSC in training data.

gene	P Value	HR (95%CI)
ITGA3	0.018	1.143 (1.023-1.277)
BIRC5	0.004	1.278 (1.080-1.513)
ITGA6	0.022	1.130 (1.018-1.255)
CDKN2A	0.019	0.953 (0.916-0.992)

Table S1-6 Univariate Cox regression analysis of differential ATGs for OS across KIRC in training data.

gene	P Value	HR (95%CI)
PTK6	3.365e-07	1.250 (1.147-1.361)
BID	1.205e-06	2.056 (1.537-2.750)
TP73	0.008	1.168 (1.041-1.311)
IFNG	0.003	1.120 (1.038-1.208)
APOL1	0.002	1.176 (1.063-1.300)
ATG16L2	0.001	1.227 (1.082-1.392)
SPHK1	1.783e-07	1.368 (1.216-1.539)
PRKCQ	0.036	0.883 (0.785-0.992)
RGS19	0.003	1.393 (1.123-1.727)
ERBB2	6.892e-05	0.733 (0.629-0.854)
BAG1	4.131e-06	0.655 (0.547-0.784)
EIF4EBP1	1.282e-07	1.510 (1.296-1.759)
BNIP3	0.001	0.810 (0.713-0.921)
MAPK10	0.0003	0.754 (0.647-0.879)

Continued Table S1-6 Univariate Cox regression analysis of differential ATGs for OS across KIRC in training data.

gene	P Value	HR (95%CI)
GAPDH	0.023	1.275 (1.033-0.572)
CASP4	0.0003	1.583 (1.232-2.033)
BIRC5	2.091e-10	1.542 (1.349-1.763)
RAB24	0.012	1.253 (1.050-1.496)
NRG3	0.027	0.882 (0.790-0.986)
P4HB	0.010	1.304 (1.065-1.597)
CDKN2A	0.001	1.280 (1.105-1.482)

Table S1-7 Univariate Cox regression analysis of differential ATGs for OS across KIRP in training data.

gene	P Value	HR (95%CI)
TP73	0.002	1.318 (1.106-1.569)
TP63	0.006	1.162 (1.044-1.292)
MYC	0.011	1.346 (1.072-1.691)
IFNG	0.0001	1.324 (1.147-1.528)
ATG16L2	0.019	0.716 (0.541-0.948)
GRID1	0.0005	1.217 (1.089-1.361)
NRG1	0.024	1.250 (1.029-1.519)
EIF4EBP1	0.0002	1.826 (1.323-2.519)
DDIT3	0.037	1.442 (1.021-2.036)
SQSTM1	0.0002	1.700 (1.281-2.255)
GAPDH	0.005	1.995 (1.239-3.215)

Continued Table S1-7 Univariate Cox regression analysis of differential ATGs for OS across KIRP in training data.

gene	P Value	HR (95%CI)
CTSD	0.010	0.695 (0.527-0.917)
BIRC5	2.844e-11	2.035 (1.651-2.509)
P4HB	0.0006	2.285 (1.423-3.670)
CDKN1A	0.003	0.700 (0.553-0.886)

Table S1-8 Univariate Cox regression analysis of differential ATGs for OS across LIHC in training data.

gene	P Value	HR (95%CI)
IKBKE	0.0006	1.222 (1.089-1.372)
TMEM74	0.010	1.135 (1.031-1.251)
GABARAPL1	0.042	0.867 (0.755-0.995)
SPHK1	0.012	1.092 (1.019-1.171)
HSPB8	0.019	1.090 (1.014-1.172)
SQSTM1	1.195e-05	1.401 (1.205-1.628)
ITGA3	0.033	1.107 (1.008-1.216)
BIRC5	1.952e-06	1.294 (1.163-1.438)
CLN3	0.002	1.405 (1.137-1.736)
RAB24	3.031e-05	1.712 (1.330-2.203)
CDKN2A	0.003	1.159 (1.052-1.277)

Table S1-9 Univariate Cox regression analysis of differential ATGs for OS across LUAD in training data.

gene	P Value	HR (95%CI)
CX3CL1	0.018	0.909 (0.839-0.984)
DLC1	0.014	0.891 (0.812-0.977)
NLRC4	0.017	0.848 (0.740-0.971)
HSPB8	0.021	0.884 (0.796-0.981)
DAPK2	3.422e-05	0.777 (0.690-0.875)
GAPDH	0.0006	1.287 (1.115-1.486)
BIRC5	0.002	1.159 (1.056-1.273)
MAP1LC3C	0.040	0.924 (0.857-0.996)
ITGB4	0.010	1.126 (1.029-1.233)

Table S1-10 Univariate Cox regression analysis of differential ATGs for OS across PAAD in training data.

gene	P Value	HR (95%CI)
PTK6	0.0004	1.290 (1.121-1.484)
TP73	0.030	1.190 (1.017-1.393)
ATG16L2	0.007	0.780 (0.650-0.935)

Table S1-11 Univariate Cox regression analysis of differential ATGs for OS across UCEC in training data.

gene	P Value	HR (95%CI)
PTK6	0.011	1.140 (1.031-1.261)
BID	0.033	1.334 (1.024-1.738)

Continued Table S1-11 Univariate Cox regression analysis of differential ATGs for OS across UCEC in training data.

gene	P Value	HR (95%CI)
MYC	0.017	1.180 (1.031-1.352)
GRID1	0.004	1.286 (1.083-1.527)
ERBB2	4.057e-05	1.313 (1.153-1.495)
EIF4EBP1	0.008	1.313 (1.072-1.609)
CDKN1B	0.049	1.174 (1.001-1.376)
BAK1	0.003	1.322 (1.096-1.594)
BIRC5	0.016	1.222 (1.039-1.438)
NRG3	1.043e-05	1.223 (1.118-1.338)
MAP1LC3C	4.632e-05	1.181 (1.090-1.280)
CDKN2A	4.008e-06	1.236 (1.129-1.352)

Table S2 Multivariate Cox regression analysis of candidate ATGs for OS across 11 cancer types in training data.

Table S2-1 Multivariate Cox regression analysis of candidate ATGs for OS across BLCA in training data.

gene	P Value	HR (95%CI)	Coefficient
IFNG	0.005	0.897 (0.832-0.968)	-0.108
APOL1	0.032	0.891 (0.802-0.990)	-0.115
SPHK1	0.002	1.169 (1.061-1.288)	0.156

Table S2-2 Multivariate Cox regression analysis of candidate ATGs for OS across BRCA in training data.

gene	P Value	HR (95%CI)	Coefficient
EIF4EBP1	0.028	1.172 (1.017-1.351)	0.159

Table S2-3 Multivariate Cox regression analysis of candidate ATGs for OS across CHOL in training data.

gene	P Value	HR (95%CI)	Coefficient
BNIP3	0.002	3.085 (1.494-6.369)	1.123

Table S2-4 Multivariate Cox regression analysis of candidate ATGs for OS across COAD in training data.

gene	P Value	HR (95%CI)	Coefficient
NRG1	0.013	0.869 (0.779-0.971)	-0.140
CDKN2A	0.037	1.136 (1.008-1.280)	0.127

Table S2-5 Multivariate Cox regression analysis of candidate ATGs for OS across HNSC in training data.

gene	P Value	HR (95%CI)	Coefficient
NKX2-3	0.026	0.939 (0.888-0.993)	-0.063

Table S2-6 Multivariate Cox regression analysis of candidate ATGs for OS across KIRC in training data.

gene	P Value	HR (95%CI)	Coefficient
ATG16L2	0.014	1.309 (1.056-1.622)	0.269
PRKCQ	0.002	0.763 (0.645-0.903)	-0.270
BAG1	0.031	0.703 (0.511-0.968)	-0.352
EIF4EBP1	0.004	1.393 (1.114-1.742)	0.331
BNIP3	0.006	0.726 (0.577-0.913)	-0.320
BIRC5	0.024	1.240 (1.029-1.496)	0.215

Table S2-7 Multivariate Cox regression analysis of candidate ATGs for OS across KIRP in training data.

gene	P Value	HR (95%CI)	Coefficient
IFNG	0.050	1.217 (1.000-1.480)	0.196
CTSD	0.004	0.527 (0.341-0.815)	-0.640
BIRC5	0.049	1.501 (1.002-2.249)	0.406
P4HB	0.002	3.214 (1.533-6.736)	1.168
CDKN1A	0.025	0.679 (0.483-0.952)	-0.388

Table S2-8 Multivariate Cox regression analysis of candidate ATGs for OS across LIHC in training data.

gene	P Value	HR (95%CI)	Coefficient
SQSTM1	0.027	1.228 (1.024-1.472)	0.205
BIRC5	0.010	1.237 (1.053-1.454)	0.213

Table S2-9 Multivariate Cox regression analysis of candidate ATGs for OS across LUAD in training data.

gene	P Value	HR (95%CI)	Coefficient
DAPK2	0.007	0.820 (0.710-0.946)	-0.199
ITGB4	0.026	1.120 (1.014-1.236)	0.113

Table S2-10 Multivariate Cox regression analysis of candidate ATGs for OS across PAAD in training data.

gene	P Value	HR (95%CI)	Coefficient
PTK6	0.005	1.267 (1.076-1.491)	0.236
ATG16L2	0.005	0.774 (0.646-0.927)	-0.257

Table S2-11 Multivariate Cox regression analysis of candidate ATGs for OS across UCEC in training data.

gene	P Value	HR (95%CI)	Coefficient
EIF4EBP1	0.006	1.426 (1.109-1.834)	0.355
NRG3	0.005	1.163 (1.046-1.294)	0.151
MAP1LC3C	0.035	1.109 (1.008-1.221)	0.104
CDKN2A	0.038	1.137 (1.007-1.283)	0.128

Table S3 Pearson correlation between ATS and the IC50 value across 7 cancer types in GDSC.

Table S3-1 Pearson correlation between ATS and the IC50 value across BLCA in GDSC.

drug	coef	pValue
AZD5438	-0.571	1.07e-02
AZD8931	0.532	1.911e-02
Bleomycin (10 uM)	-0.579	1.182e-02
Bleomycin (50 uM)	-0.566	1.426e-02
FEN1_3940	-0.631	3.796e-03
Fulvestrant	-0.664	3.649e-03
IC-87114	-0.649	2.628e-03
IMD-0354	-0.515	2.418e-02
kb NB 142-70	-0.535	1.822e-02
KIN001-270	-0.516	2.374e-02
Kobe2602	-0.586	8.42e-03
KU-55933	-0.554	1.391e-02
MCT1_6447	-0.647	2.76e-03
MetAP2 Inhibitor, A832234	-0.503	2.826e-02
PARP_9495	-0.542	2.014e-02
Ponatinib	0.527	2.056e-02
RO-3306	-0.513	2.484e-02
Selisistat	-0.575	9.987e-03
T0901317	-0.525	2.109e-02
TW 37	-0.565	1.175e-02

Continued Table S3-1 Pearson correlation between ATS and the IC50 value across BLCA in GDSC.

drug	coef	pValue
XMD8-92	-0.802	3.018e-02
YK-4-279	-0.593	1.206e-02
Zibotentan	-0.54	1.702e-02

Table S3-2 Pearson correlation between ATS and the IC50 value across HNSC in GDSC.

drug	coef	pValue
(5Z)-7-Oxozeaenol	-0.549	4.367e-04
QL-VIII-58	-0.738	1.09e-03
XMD11-85h	-0.559	2.433e-02

Table S3-3 Pearson correlation between ATS and the IC50 value across KIRC in GDSC.

drug	coef	pValue
AS601245	0.611	1.524e-03
BAY-61-3606	0.533	8.794e-03
BMS-754807	0.51	1.089e-02
Crizotinib	-0.609	4.674e-02
HG-5-88-01	0.543	1.998e-02
Lapatinib	0.716	2.999e-02
NVP-TAE684	0.628	3.857e-02
Sorafenib	0.633	4.967e-02

Table S3-4 Pearson correlation between ATS and the IC50 value across LIHC in GDSC.

drug	coef	pValue
ACY-1215	0.522	3.175e-02
AICA Ribonucleotide	-0.706	2.254e-03
GW-2580	-0.647	4.991e-03
JAK3_7406	-0.668	4.676e-03
Palbociclib	-0.523	3.784e-02
PD0325901	0.56	2.411e-02
Refametinib	0.691	1.677e-05
Trametinib	0.72	1.653e-03

Table S3-5 Pearson correlation between ATS and the IC50 value across LUAD in GDSC.

drug	coef	pValue
JW-7-52-1	0.627	3.907e-02
Salubrinal	0.577	4.957e-02

Table S3-6 Pearson correlation between ATS and the IC50 value across PAAD in GDSC.

drug	coef	pValue
Amuvatinib	0.51	4.751e-03
Etoposide	0.503	4.571e-03

Table S3-7 Pearson correlation between ATS and the IC50 value across UCEC in GDSC.

drug	coef	pValue
ARRY-520	0.686	4.127e-02

Continued Table S3-7 Pearson correlation between ATS and the IC50 value across UCEC in GDSC.

drug	coef	pValue
AT7867	0.777	1.374e-02
AZD7762	0.618	6.233e-03
BMS-754807	0.704	3.43e-02
Capivasertib	0.75	1.996e-02
Doxorubicin	0.51	3.079e-02
Flavopiridol	0.709	3.238e-02
Midostaurin	0.708	3.278e-02
PD0325901	0.731	2.535e-02
Refametinib	0.621	7.766e-03
RO-3306	0.862	2.813e-03
Temsirolimus	0.667	4.99e-02
Trichostatin A	0.771	1.508e-02
Vinorelbine	0.689	3.993e-02
ZM447439	0.74	2.265e-02

Table S4 Pearson correlation between ATS and the AUC value across 7 cancer types in GDSC.

Table S4-1 Pearson correlation between ATS and the AUC value across BLCA in GDSC.

drug	coef	pValue
AZD5438	-0.562	1.229e-02
AZD8931	0.596	7.091e-03
Bleomycin (10 uM)	-0.568	1.389e-02
Bleomycin (50 uM)	-0.577	1.217e-02
Dactolisib	-0.504	2.796e-02
FEN1_3940	-0.612	5.338e-03
FGFR_0939	0.569	1.096e-02
Fulvestrant	-0.536	2.641e-02
IMD-0354	-0.516	2.384e-02
Ponatinib	0.59	7.832e-03
Quizartinib	0.676	1.481e-03
TW 37	-0.547	1.542e-02
VNLG124	0.598	6.84e-03
YK-4-279	-0.584	1.387e-02

Table S4-2 Pearson correlation between ATS and the AUC value across HNSC in GDSC.

drug	coef	pValue
(5Z)-7-Oxozeaenol	-0.505	1.45e-03
PI3Ka_4409	-0.537	3.577e-04
QL-VIII-58	-0.749	8.363e-04
QL-XII-61	-0.552	3.307e-02

Continued Table S4-2 Pearson correlation between ATS and the AUC value across HNSC in GDSC.

drug	coef	pValue
Salubrinal	0.967	3.299e-02
XMD11-85h	-0.564	2.286e-02

Table S4-3 Pearson correlation between ATS and the AUC value across KIRC in GDSC.

drug	coef	pValue
AS601245	0.672	3.228e-04
BMS-754807	0.551	5.234e-03
Lapatinib	0.725	2.707e-02
NVP-TAE684	0.611	4.575e-02

Table S4-4 Pearson correlation between ATS and the AUC value across LIHC in GDSC.

drug	coef	pValue
AICA Ribonucleotide	-0.701	2.496e-03
eEF2K Inhibitor, A-484954	-0.538	3.164e-02
GW-2580	-0.647	5.034e-03
JAK3_7406	-0.737	1.127e-03
Refametinib	0.634	1.264e-04
Selisistat	-0.575	1.566e-02
Serdemetan	-0.568	2.173e-02
STF-62247	-0.55	2.232e-02
THZ-2-49	-0.523	3.773e-02

Continued Table S4-4 Pearson correlation between ATS and the AUC value across LIHC in GDSC.

drug	coef	pValue
Trametinib	0.691	3.033e-03
UNC1215	-0.551	2.692e-02

Table S4-5 Pearson correlation between ATS and the AUC value across LUAD in GDSC.

drug	coef	pValue
XMD8-85	-0.69	1.299e-02

Table S4-6 Pearson correlation between ATS and the AUC value across PAAD in GDSC.

drug	coef	pValue
Daporinad	0.529	4.56e-03
Ispinesib Mesylate	0.505	4.446e-03

Table S4-7 Pearson correlation between ATS and the AUC value across UCEC in GDSC.

drug	coef	pValue
AT7867	0.688	4.06e-02
Capivasertib	0.705	3.397e-02
Doxorubicin	0.509	3.104e-02
FGFR_0939	-0.696	3.713e-02
Flavopiridol	0.673	4.675e-02
Refametinib	0.524	3.09e-02
RO-3306	0.794	1.056e-02
SGC0946	-0.714	3.081e-02

Continued Table S4-7 Pearson correlation between ATS and the AUC value across UCEC in GDSC.

drug	coef	pValue
Trichostatin A	0.764	1.655e-02
Vinorelbine	0.673	4.715e-02
ZM447439	0.716	3e-02
